# Evaluation of symptomatic maxillary sinus pathologies using panoramic radiography and cone beam computed tomography—influence of professional training

**DOI:** 10.1186/s40729-017-0075-5

**Published:** 2017-04-05

**Authors:** Michael Dau, Paul Marciak, Bial Al-Nawas, Henning Staedt, Abdulmonem Alshiri, Bernhard Frerich, Peer Wolfgang Kämmerer

**Affiliations:** 1 Department of Oral, Maxillofacial and Plastic Surgery, University Medical Center, Schillingallee 35, 18057 Rostock, Germany; 2 Department of Oral and Maxillofacial Surgery, Plastic Surgery, University Medical Centre, Mainz, Germany; 3 Private Dental Praxis Dr. Rossa, Ludwigshafen, Germany; 4 Department of Biomaterial and Prosthetic Sciences, King Saud University, Riyadh, Saudi Arabia

**Keywords:** Panoramic radiography, Cone beam computed tomography, Maxillary sinus site, Subjective rating, Incidental radiographic findings, Education

## Abstract

**Background:**

A comparison of panoramic radiography (PAN) alone and PAN together with small field of view cone beam computed tomography (sFOV-CBCT) for diagnosis of symptomatic pathologies of the maxillary sinus was carried out by clinicians of different experience.

**Methods:**

Corresponding radiographic images (PAN/sFOV-CBCT) of 28 patients with symptomatic maxillary sinus pathologies were chosen and analyzed by two general practitioners (GP), two junior maxillofacial surgeons (MS1), and three senior maxillofacial surgeons (MS2) via questionnaire.

**Results:**

Visibility of maxillary pathologies in PAN was significantly different between the groups (GP 39%, MS1 48%, MS2 61%; *p* < 0.05). The number of incidental findings varied within examiner groups in PAN with a significant increase in MS2 (*p* = 0.027). The majority of examiners rated an additional sFOV-CBCT as “reasonable”/“required” with a significant influence of the examining groups (GP 98.2%, MS1 94.6%, MS2 80.9%; *p* = 0.008). In 58% of cases, an additional sFOV-CBCT was seen as “affecting therapy” with significant differences between the groups (GP 68%, MS1 50%, MS2 55%; *p* < 0.001).

**Conclusions:**

PAN alone is not sufficient for the evaluation of pathologies of the maxillary sinus. But, depending on the examiners’ clinical experience, it remains a useful diagnostic tool. Along with the observers’ training, significant benefits of an additional sFOV-CBCT for evaluation of symptomatic maxillary sinus pathologies were detected.

## Background

Non-symptomatic abnormalities of the maxillary sinus such as mucosal thickening, retention cysts, and opacification are reported to occur in up to 74% of all cases [1–6]. For diagnosis of symptomatic pathologies of the maxillary sinus like retention cysts, polyps, and tumors, panoramic radiographies (PAN) are commonly used and widely available. In PAN, not every area of interest is accurately detected and allocated. Furthermore, small maxillary sinus lesions with diameter less than 3 mm show poor detection rates [7]. Three-dimensional imaging is useful in the maxilla for a wide range of clinical settings, such as trauma, bone pathology, and neoplastic diseases, as well as in dental implantology and sinus augmentation [8–12].

Computed tomography (CT) is an excellent tool for maxillary sinus examination and diagnosis [13, 14]. A survey among 331 otolaryngologists showed that the majority (75%) did not obtain confirmatory CT scan before initial non-surgical therapy. Though, prior proceeding with sinus surgery, an average of one (59%) or even two (37%) CT scans was reported [15]. Cone beam computed tomography (CBCT) is mostly used for dental implant planning [6, 10, 16] and offers diagnostic options similar to CT scans but without contrast agents and with about 10–50% less radiation exposure [17, 18]. Especially if small fields of view are used for CBCT, radiation exposure is significantly reduced. However, this exposure to radiation as well as the costs are still significantly higher when compared to those of conventional dental imaging [19–22]. For diagnosis and general preoperative planning, both PAN and CBCT are described to be useful and important diagnostic tools [11, 23, 24]. Nonetheless, there are only few studies [7, 12, 14, 25] and some case reports [26–28] that showed an additional clinical benefit of CBCT for evaluation of maxillary sinus when compared to PAN. In most studies, non-symptomatic sites were visualized in order to exclude pathologic findings prior to dental implant surgery [1, 7, 23, 24, 29, 30].

In order to justify CBCT use for clinical examination and diagnosis of the maxillary sinus, the aim of this study was to compare the subjective quality rating of PAN and PAN together with a small field of view (sFOV) CBCT to evaluate symptomatic maxillary sinus by clinicians with different training and clinical experience.

## Methods

### Patients and examiners

In an experimental diagnostic comparison, radiographic images of 15 female and 13 male patients were assessed. Patients’ radiographs were selected from the Department of Oral, Maxillofacial and Facial Plastic Surgery of the University Medical Centre of Mainz and Rostock, Germany. All patients have had referrals to the hospitals with symptomatic maxillary sinus pathologies and received PAN (Orthophos XG Plus (Sirona Dental Systems GmbH, Bensheim, Germany)) as well as CBCT (KaVo 3D eXam, KaVo Dental GmbH, Biberach/Riß, Germany or Accuitomo Morita, J. MORITA Mfg. Corp., Kyoto, Japan) for radiographic analysis and diagnosis. All CBCT images contained a limited field of view (size of FOV 60 × 60 mm) for the pathological site only (sFOV-CBCT). Clinical information were given to all examiners before rating. Patients with incomplete medical records were excluded. Seven examiners with a different professional training and experience in using PAN and CBCT were participating. They were two general practitioners with 2–3 years of clinical experience (GP), two junior maxillofacial surgeons with 2–3 years of clinical experience (MS1), and three senior maxillofacial surgeons with 6–7 years of clinical experience (MS2). A standardized questionnaire for PAN (three questions) and CBCT (two questions) was given to each individual separately to answer. The participant examined only PAN in the first part of the project. Afterwards, he/she filled out the respective questionnaire. At the next step, he/she examined the CBCT scans and answered its related questions. All examiners had undergone a structured postgraduate curriculum for usage of CBCT before, and they used CBCT on daily basis. This curriculum, as demanded by German authorities for using CBCT, consisted of at least two classroom-based trainings (each for 1 day) together with 25 documented CBCT cases and a written examination. Besides, there was no further training for this study. In each case, the same reading environment using a beamer (Epson® EB G5450WU, Epson® Germany, Meerbusch, Germany; data sheet: resolution 1920 × 1200, brightness 4000 lumens, contrast ratio 1000:1) and a 2 × 3 m screen was provided. This study on anonymous radiographic images was performed in accordance to the current version of the Declaration of Helsinki [31].

### Questionnaire

The first question for PAN addressed the imaging quality in the clinical relevant area of interest (clinical data were given). Three answers were possible: 1 = good visibility and can be evaluated, 2 = visible but cannot be evaluated, and 3 = not visible. The second question asked for an additional need for CBCT scans. Three answers were possible: 1 = required, 2 = reasonable, and 3 = not required. The third question was referring to the number of additional incidental findings in PAN not related to the sinus disease that led to the radiographic examination.

For CBCT, the first question was referring to a possible additional value in the area of interest. The examiners had to choose between three possible answers (1 = showed no additional information, 2 = was useful, 3 = was affecting therapy). The second question targeted the number of incidental findings in CBCT in addition to PAN not related to the sinus disease that led to the radiographic examination.

### Statistics

Due to the experimental design, no prior power analysis was conducted. All results in this study were expressed as number of cases, incidence value (percentage), or as arithmetic means ± standard deviation (SD). For comparison of groups, one-way analyses of variance (ANOVA) with Tukey B simultaneous post hoc tests as well as chi-square tests were performed and descriptive *p* values of the tests are reported. A *p* value ≤0.05 was termed significant. All statistical analyses were performed with SPSS version 20.0 (SPSS Inc., Chicago, IL, USA).

## Results

**Fig. 1:**
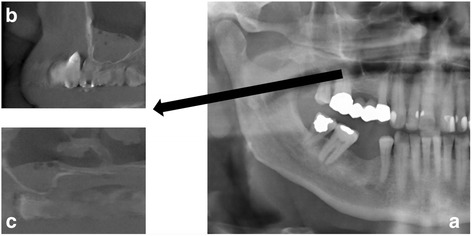
**a** Panoramic radiography with area of interest (maxillary sinus) and **b**, **c** examples of corresponding images in cone beam computed tomography

**Fig. 2:**
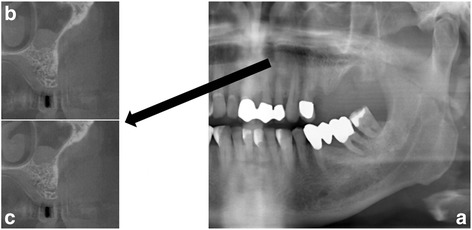
**a** Panoramic radiography with area of interest (maxillary sinus) and **b**, **c** examples of corresponding images in cone beam computed tomography

This study focused on three different aspects in our analysis—PAN, PAN and CBCT, as well as the influence of the different clinical and radiological experience (examples in Figs. 1 and 2).

- Panoramic radiography (PAN)

When assessing PAN, the ratings were significantly lower at “good visible and can be evaluated” (9.9%) compared to “visible but cannot be evaluated” (39.5%; *p* < 0.001) and compared to “not visible” (50.6%; *p* < 0.001) ratings (Table 1). An additional CBCT was needed in most cases (“required” (28%) and “reasonable” (63.3%) versus “not required” (8.7%; Table 2)). All examiners found an average number of 1.7 ± 1.3 additional findings in PAN (Table 3). The three most common findings were retained third molars with putative follicular cysts (22% of all findings), followed by radiological insufficient root filling (21%) and caries/insufficient filling of teeth (19%; Table 4).

**Table 1:** Results of the question “Based on PAN, the clinical area of interest, is…”

Question	General practitioner (*n* = 2)	Junior maxillofacial surgeon (*n* = 2)	Senior maxillofacial surgeon (*n* = 3)	*p* value*
1 = good visibility and can be evaluated	3 (5.4%)	5 (8.9%)	13 (15.5%)	*p* < 0.002
2 = visible but cannot be evaluated	19 (33.9%)	22 (39.3%)	38 (45.2%)	
3 = not visible	34 (60.7%)	29 (51.8%)	33 (39.3%)	

*One-way ANOVA test with post hoc Tukey B: GP versus MS1 *p* = 0.034, MS1 versus MS2 *p* = 0.211, and GP versus MS2 *p* = 0.001

**Table 2:** Results of the question “An additional sFOV-CBCT of the clinical area of interest is…”

Question	General practitioner (*n* = 2)	Junior maxillofacial surgeon (*n* = 2)	Senior maxillofacial surgeon (*n* = 3)	*p* value*
1 = required	14 (25.0%)	21 (37.5%)	18 (21.4%)	*p* = 0.008
2 = reasonable	41 (73.2%)	32 (57.1%)	50 (59.5%)	
3 = not required	1 (1.8%)	3 (5.4%)	16 (19.0%)	

*One-way ANOVA test with post hoc Tukey B: GP versus MS1 *p* = 0.369, MS1 versus MS2 *p* = 0.006, and GP versus MS2 *p* = 0.038

**Table 3:** Number of additional incidental findings in PAN and sFOV-CBCT not related to the sinus disease that led to the radiographic examination

Number of cases	General practitioner (*n* = 2)	Junior maxillofacial surgeon (*n* = 2)	Senior maxillofacial surgeon (*n* = 3)	*p* value*
	Number of incidental findings in PAN			
(*n* = 28)	1.5 ± 1.3	1.6 ± 1.1	2.1 ± 1.5	*p* = 0.027
	Number of additional, incidental findings in sFOV-CBCT			
(*n* = 28)	0.7 ± 0.5	0.6 ± 0.5	0.7 ± 0.7	*p* = 0.912

*One-way ANOVA test

**Table 4:** Description of incidental findings in PAN not related to the sinus disease that led to the radiographic examination

Additional incidental findings in panoramic radiography	Relative incidence (%) in relation to total number of therapy affecting findings
Retained third molar/follicular cyst	22
Insufficient root filling	21
Caries/insufficient filling of teeth	19
Apical ostitis	17
Remaining root remnants	9
Periodontal bone loss	8
Anatomic particularities (enlargement of the mental foramen/retromolar foramen/bifid nerve)	4

(b) Cone beam computed tomography (CBCT)

The majority of the answers indicated the usefulness of an additional sFOV-CBCT. Whereas it only “showed no additional information” in 10.1% and was “useful” in 32.4% of cases, an additional CBCT was rated as “affecting therapy” in 57.5% of cases (Table 5).

**Table 5:** Results of the question “Is there an additional clinical value of sFOV-CBCT?”

Question	General practitioner (*n* = 2)	Junior maxillofacial surgeon (*n* = 2)	Senior maxillofacial surgeon (*n* = 3)	*p* value*
1 = it showed no additional information	2 (3.6%)	5 (8.9%)	15 (17.9%)	*p* < 0.001
2 = it was useful	16 (28.6%)	23 (41.1%)	23 (27.4%)	
3 = it was affecting therapy	38 (67.8%)	28 (50.0%)	46 (54.8%)	

*One-way ANOVA test with post hoc Tukey B: GP versus MS1 *p* = 0.002, MS1 versus MS2 *p* = 0.890, and GP versus MS2 *p* = 0.001

Overall, the examiners observed an average number of 0.6 ± 0.6 additional incidental findings (Table 3). The findings were radiological caries/insufficient filling of teeth (88%) as well as insufficient root filling (22%).

(c) Influence of examiners’ clinical background

In PAN, MS1 (51.8%) and MS2 (39.3%) rated significantly less for “not visible” when compared to GPs (60.7%; *p* < 0.001). The difference was significant between MS1 and MS2 as well (*p* < 0.05). Significantly more “good visibility” ratings were obtained for MS2 (15.5%) when compared to MS1 (8.9%; *p* = 0.021) and GP (5.4%; *p* < 0.001; Table 1). A significant higher number of additional incidental findings in PAN was seen in MS2 (mean = 2.1 ± 1.5) versus GP (mean = 1.5 ± 1.3; *p* = 0.021) as well as in MS2 versus MS1 (mean = 1.6 ± 1.1; *p* = 0.048; Table 3).

GPs rated an additional CBCT significantly less often to be “not required” (1.8%) when compared to MS1 (5.4%; *p* = 0.038) and to MS2 (19%; *p* = 0.006). Moreover, GPs rated significantly more for a CBCT to be “reasonable” or “required” (98.2%) when compared to MS1 (94.6%; *p* = 0.002) and compared to MS2 (80.9%; *p* = 0.001; Table 2). Also, in the GP group, the additional CBCT was seen significantly more often to be “affecting therapy” (67.8%) when compared to MS1 (50%) and to MS2 (53.8%; all *p* < 0.001; Table 5). Between the groups, there was no difference in the average number of additional incidental diagnoses in sFOV-CBCT scans (GD, average = 0.7 ± 0.5; MS1, average = 0.6 ± 0.5; MS2, average = 0.7 ± 0.7; *p* = 0.912, Table 3).

## Discussion

In dentistry, PAN is a widely available, useful, and important diagnostic tool for diagnosis and general preoperative planning [32] with less radiation exposure then CBCT [21]. While most dentists have used it routinely successful for years and gained significant experience in doing so [33], there are certain limitations in dependence of the region to be examined [10]. The high number of “not visible” ratings of the area of interest in the study at hand underlines this conclusion. Nevertheless, PAN showed several additional incidental findings showing its important value being a basic diagnostic tool, also in preventive dentistry [32, 33]. It is noticeable that most of these incidental findings were described by senior surgeons. This demonstrates the impact of clinical experience of evaluation of PAN [12, 34]. A lack of experience in 2D imaging might even result in unnecessary additional 3D diagnostics (such as additional CBCTs) [12].

For the diagnosis of symptomatic pathologies in the maxillary sinus, PAN alone is not sufficient. Benefits (in dependence of clinical and radiological experience) offered from additional sFOV-CBCT imaging were proven in the presented study. The high number of “therapy affecting” ratings when adding CBCT supports such statement. Wolf et al. reported the general demand for three-dimensional imaging of maxillary sinus in order to minimize intra- and postoperative complications and to localize any foreign body in relation to other anatomical structures [35]. Similarly, various studies reported an average of one or more CT scans prior proceeding with sinus surgery [15]. Sharma et al. recommended CT scans prior sinus surgery in order to guide the surgeon [36]. Other researcher found the same diagnostic accuracy of CBCT scans of maxillary sinus pathologies when compared to sinus endoscopy [37] which underlines the importance of CBCT within this field. As shown by others as well [2, 38–43], a better evaluation of anatomical structures was found when using CBCT. CBCT scans offer an extremely valuable diagnostic and clinical tool for maxillary sinus pathologies in general [36, 44, 45] for vital findings like posterior superior alveolar arteries in the lateral sinus wall [46] as well as for anatomical variations [47]. Especially in cases with symptomatic maxillary sinus pathologies, three-dimensional diagnostic is helpful [13, 36, 48] and a sFOV-CBCT offers limited radiation exposure as well.

The influence of clinical experience of evaluation of PAN [34] as well as the clinical experience and routine analysis of 3D radiographs (as assumed for maxillofacial surgeons when compared to those for general practitioners) strongly influence the diagnostic value of additional three-dimensional imaging. The number of incidental findings in CBCT in addition to those seen in PAN was not of major difference and did not correlate to the examiners’ experience. This difference can be explained by using sFOV-CBCTs for evaluation of symptomatic maxillary sinus pathologies only. The smaller field of view shows less incidental findings, but the radiation exposure will be kept lower as well. Nonetheless, sFOV-CBCT is not meant to be a replacement for PAN especially for patients’ screening. It seems that advanced diagnostic tools such as CBCT offer an effective solution with more precise diagnosis of the maxillary sinus when compared to PAN together with a lower radiation dose compared to a CT.

There are some limitations of the study and potential bias caused by the experimental design, the subjective evaluation, and the low number of patients. Nevertheless, the additional value of CBCT strongly depends on the level on medical and radiographic knowledge of the anatomy of sites of interest [16, 38] and the surrounding structures [2]. Based on the findings of this study and the literature, an adjunct sFOV-CBCT is a valuable diagnostic tool for cases of symptomatic maxillary sinus pathologies.

## Conclusions

Depending on the observers’ clinical and radiological experience, PAN alone may not be sufficient for evaluation of pathologies of the maxillary sinus. On the contrary, significant benefits of sFOV-CBCT for diagnosing symptomatic maxillary sinus pathologies were reported. Having sFOV-CBCT seems to have added additional information and confidence in comparison to PAN alone. Nonetheless, also with the examiners’ increased clinical experience, PAN remains a valuable diagnostic tool.

## References

[1] Dragan E (2014). Maxillary sinus anatomic and pathologic CT findings in edentulous patients scheduled for sinus augmentation. Rev Med Chir Soc Med Nat Iasi.

[2] Raghav M (2014). Prevalence of incidental maxillary sinus pathologies in dental patients on cone-beam computed tomographic images. Contemp Clin Dent.

[3] Lyros I (2014). An incidental finding on a diagnostic CBCT: a case report. Aust Orthod J.

[4] Steier L (2014). Maxillary sinus unilateral aplasia as an incidental finding following cone-beam computed (volumetric) tomography. Aust Endod J.

[5] Vogiatzi T (2014). Incidence of anatomical variations and disease of the maxillary sinuses as identified by cone beam computed tomography: a systematic review. Int J Oral Maxillofac Implants.

[6] Warhekar S (2015). Incidental findings on cone beam computed tomography and reasons for referral by dental practitioners in Indore City (m.p). J Clin Diagn Res.

[7] Shiki K (2014). The significance of cone beam computed tomography for the visualization of anatomical variations and lesions in the maxillary sinus for patients hoping to have dental implant-supported maxillary restorations in a private dental office in Japan. Head Face Med.

[8] Dammann F (2014). Diagnostic imaging modalities in head and neck disease. Dtsch Arztebl Int.

[9] Kuhnel TS, Reichert TE (2015). Trauma of the midface. Laryngorhinootologie.

[10] Dau M (2017). Presurgical evaluation of bony implant sites using panoramic radiography and cone beam computed tomography-influence of medical education. Dentomaxillofac Radiol.

[11] Kammerer PW (2016). Surgical evaluation of panoramic radiography and cone beam computed tomography for therapy planning of bisphosphonate-related osteonecrosis of the jaws. Oral Surg Oral Med Oral Pathol Oral Radiol.

[12] Malina-Altzinger J (2015). Evaluation of the maxillary sinus in panoramic radiography—a comparative study. Int J Implant Dent.

[13] Guerra-Pereira I., et al. Ct maxillary sinus evaluation—a retrospective cohort study. Med Oral Patol Oral Cir Bucal. 2015;20(4):e419–26. 10.4317/medoral.20513 PMC4523254 25858084

[14] Gang TI (2014). The effect of radiographic imaging modalities and the observer’s experience on postoperative maxillary cyst assessment. Imaging Sci Dent.

[15] Batra PS (2015). Computed tomography imaging practice patterns in adult chronic rhinosinusitis: survey of the American Academy of Otolaryngology-Head and Neck Surgery and American Rhinologic Society Membership. Int Forum Allergy Rhinol.

[16] Whitesides LM, Aslam-Pervez N, Warburton G (2015). Cone-beam computed tomography education and exposure in oral and maxillofacial surgery training programs in the United States. J Oral Maxillofac Surg.

[17] Shah N, Bansal N, Logani A (2014). Recent advances in imaging technologies in dentistry. World J Radiol.

[18] De Cock J (2015). A comparative study for image quality and radiation dose of a cone beam computed tomography scanner and a multislice computed tomography scanner for paranasal sinus imaging. Eur Radiol.

[19] Roberts JA (2009). Effective dose from cone beam CT examinations in dentistry. Br J Radiol.

[20] Deman P (2014). Dose measurements for dental cone-beam CT: a comparison with MSCT and panoramic imaging. Phys Med Biol.

[21] Shin HS (2014). Effective doses from panoramic radiography and CBCT (cone beam CT) using dose area product (DAP) in dentistry. Dentomaxillofac Radiol.

[22] Al-Okshi A (2013). Using GafChromic film to estimate the effective dose from dental cone beam CT and panoramic radiography. Dentomaxillofac Radiol.

[23] Poleti ML (2014). Anatomical variation of the maxillary sinus in cone beam computed tomography. Case Rep Dent.

[24] Friedland B, Metson R (2014). A guide to recognizing maxillary sinus pathology and for deciding on further preoperative assessment prior to maxillary sinus augmentation. Int J Periodontics Restorative Dent.

[25] Agacayak KS (2015). Alterations in maxillary sinus volume among oral and nasal breathers. Med Sci Monit.

[26] Jafari-Pozve N (2014). Aplasia and hypoplasia of the maxillary sinus: a case series. Dent Res J (Isfahan).

[27] Rivis M, Valeanu AN (2013). Giant maxillary cyst with intrasinusal evolution. Rom J Morphol Embryol.

[28] Yilmaz SY, Misirlioglu M, Adisen MZ (2014). A diagnosis of maxillary sinus fracture with cone-beam CT: case report and literature review. Craniomaxillofac Trauma Reconstr.

[29] Lana JP (2012). Anatomic variations and lesions of the maxillary sinus detected in cone beam computed tomography for dental implants. Clin Oral Implants Res.

[30] Jadhav AB, Lurie AG, Tadinada A (2014). Chronic osteitic rhinosinusitis as a manifestation of cystic fibrosis: a case report. Imaging Sci Dent.

[31] World Medical A (2013). World Medical Association Declaration of Helsinki: ethical principles for medical research involving human subjects. JAMA.

[32] Kammerer PW (2016). Clinical parameter of odontoma with special emphasis on treatment of impacted teeth—a retrospective multicentre study and literature review. Clin Oral Investig.

[33] Schafer T (2015). Incidental finding of a foreign object on a panoramic radiograph. Pediatr Dent.

[34] Turgeon DP, Lam EW (2016). Influence of experience and training on dental students’ examination performance regarding panoramic images. J Dent Educ.

[35] Wolf MK, et al. Preoperative 3D imaging in maxillary sinus: brief review of the literature and case report. Quintessence Int. 2015;46(7):627–31. 10.3290/j.qi.a33930 25918753

[36] Sharma BN (2015). Computed tomography in the evaluation of pathological lesions of paranasal sinuses. J Nepal Health Res Counc.

[37] Zojaji R (2015). Diagnostic accuracy of cone-beam computed tomography in the evaluation of chronic rhinosinusitis. ORL J Otorhinolaryngol Relat Spec.

[38] Noar JH, Pabari S (2013). Cone beam computed tomography—current understanding and evidence for its orthodontic applications?. J Orthod.

[39] Guerrero ME, Noriega J, Jacobs R (2014). Preoperative implant planning considering alveolar bone grafting needs and complication prediction using panoramic versus CBCT images. Imaging Sci Dent.

[40] Machtei EE, Oettinger-Barak O, Horwitz J (2014). Axial relationship between dental implants and teeth/implants: a radiographic study. J Oral Implantol.

[41] Stratemann SA (2010). Evaluating the mandible with cone-beam computed tomography. Am J Orthod Dentofacial Orthop.

[42] Quintero JC (1999). Craniofacial imaging in orthodontics: historical perspective, current status, and future developments. Angle Orthod.

[43] Tadinada A (2015). Radiographic evaluation of the maxillary sinus prior to dental implant therapy: a comparison between two-dimensional and three-dimensional radiographic imaging. Imaging Sci Dent.

[44] Ritter L (2011). Prevalence of pathologic findings in the maxillary sinus in cone-beam computerized tomography. Oral Surg Oral Med Oral Pathol Oral Radiol Endod.

[45] Maillet M (2011). Cone-beam computed tomography evaluation of maxillary sinusitis. J Endod.

[46] Varela-Centelles P (2015). Detection of the posterior superior alveolar artery in the lateral sinus wall using computed tomography/cone beam computed tomography: a prevalence meta-analysis study and systematic review. Int J Oral Maxillofac Surg.

[47] Shahidi S (2016). Evaluation of anatomic variations in maxillary sinus with the aid of cone beam computed tomography (CBCT) in a population in south of Iran. J Dent (Shiraz).

[48] Hssaine K (2016). Paranasal sinus mucoceles: about 32 cases. Rev Stomatol Chir Maxillofac Chir Orale.

